# Coronary-Cameral Fistula with Angina Pectoris

**DOI:** 10.1155/2010/362532

**Published:** 2010-12-06

**Authors:** Mehmet Akif Cakar, Ersan Tatli

**Affiliations:** ^1^Cardiology Department, Sakarya Education and Research Hospital, Korucuk, 54100 Sakarya, Turkey; ^2^Cardiology Department, Ada Tip Private Hospital, 54100 Sakarya, Turkey

## Abstract

Coronary-cameral fistula (CCF) is an anomalous connection between a coronary artery and cardiac chamber. Most of CCFs are discovered incidentally during angiographic evaluation for coronary vascular disorder. We report a case of CCF with angina pectoris. Selective coronary arteriography revealed diffuse CCF involving the left anterior descending artery (LAD) emptying into left ventricle (LV) and showed significant two-vessel coronary artery stenosis.

## 1. Introduction

 CCF is a rare condition in which a communication exists between a coronary artery and a cardiac chamber. Most of CCFs are discovered incidentally during angiographic evaluation for coronary vascular disorder. Although most patients are asymptomatic, it can lead to symptoms of angina pectoris [1]. We report a case of CCF with angina pectoris. Selective coronary arteriography revealed diffuse CCF involving the (LAD) emptying in to left ventricle (LV) and showed significant two-vessel coronary artery stenosis including LAD, circumflex artery (Cx).

## 2. Case Presentation

**Figure 1:**
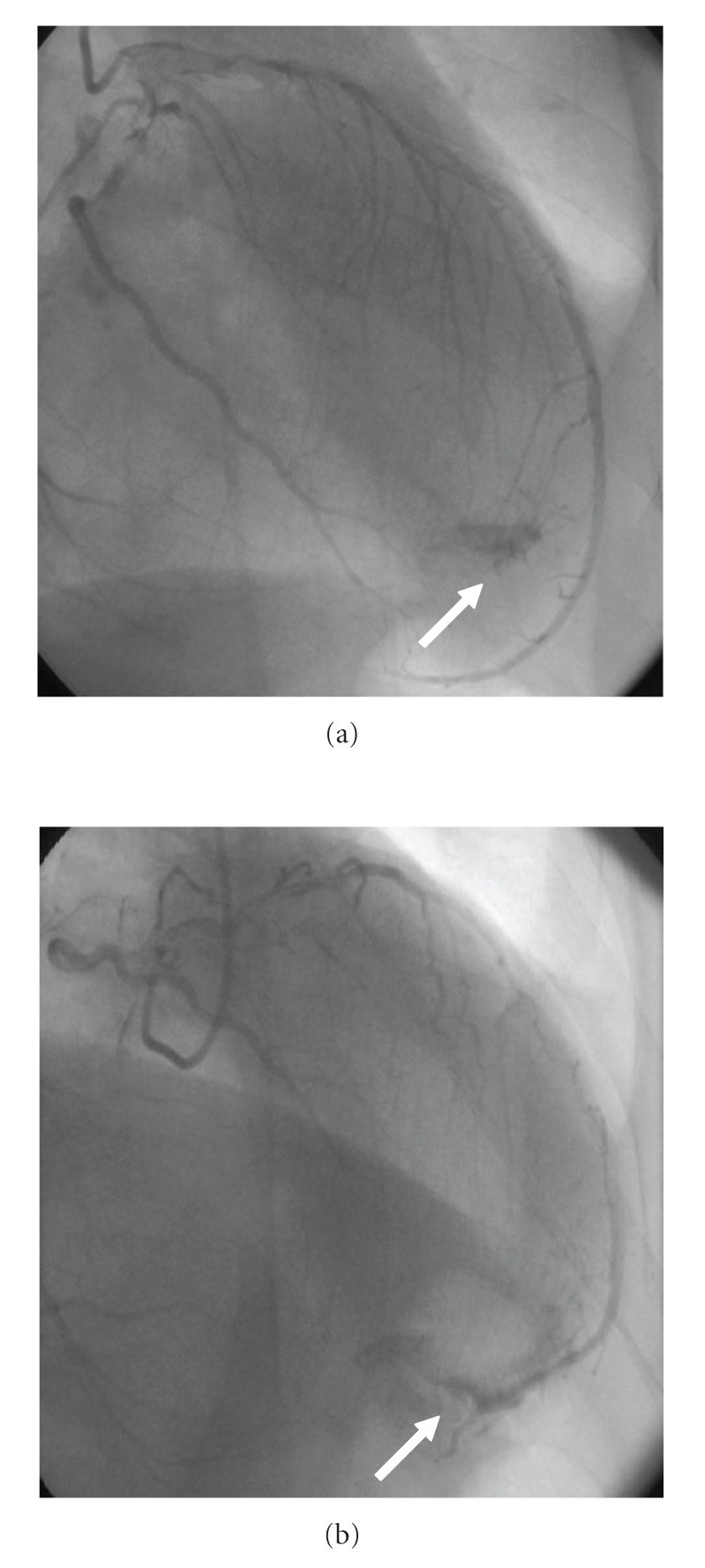
Figures (a) and (b) Fistula from distal of left coronary artery to the left ventricle is evident.

 A 59-year-old man was admitted to our hospital complaining of anterior chest pain on exertion. He had a history of hypertension and diabetes. His blood pressure was 130/80 mmHg, and the pulse rate was 72 beats/minute. There was no audible murmur on the chest wall, and his electrocardiography (ECG) was normal. His exercise treadmill test (ETT) revealed ischemic changes accompanied by chest pain. Therefore, selective coronary angiography was performed via the right femoral approach (Seldinger technique). Coronary angiography showed critical lesion in LAD, Cx, and the contrast agent entered the left ventricle from the left anterior descending artery (LAD) during diastole (see Figures 1(a) and 1(b)). We performed successful percutaneous coronary intervention and stenting to lesion in LAD and Cx. To our knowledge, this is the first case reported in the literature. 

The patient's post-PTCA course was uneventful with the disappearance of angina and symptoms. One month after discharge, ETT was performed to the patient and demonstrated no ischemic ECG changes.

## 3. Discussion

 Coronary artery fistulas are communications between one of the coronary arteries and a cardiac chamber (CCF) or a major vessel (venae cavae, pulmonary artery, veins, or coronary sinus). CCFs are seen in 0.1% of patients undergoing coronary angiograms. Major sites of origin of fistula are the right coronary artery (55%), left coronary artery (35%), and both coronary arteries (5%). Major termination sites are the right ventricle (40%), right atrium (26%), pulmonary arteries (17%) and less frequently the superior vena cava or coronary sinus and least often the left atrium and left ventricle. Coronary artery-left ventricular fistulae are exceedingly rare with the incidence being reported as 1.2% of all coronary artery fistulae [2]. Cardiac catheterization with coronary angiography remains the gold standard for the diagnosis of coronary artery fistula. It can demonstrate the size, anatomy, number, origination, and termination site of the fistulas. Cardiac echocardiography is also useful for diagnosis. Magnetic resonance imaging and multidetector computed tomography are also used to evaluate the anatomy, flow, and function of CCF [3–5].

 In some cases, fistula can cause ischemia by coronary artery steal phenomenon which leads to ischemia of the segment of the myocardium perfused by the coronary artery [1, 6]. Kwang Kon Koh et al. demonstrated myocardial ischemia on treadmill test and Holter monitoring in patients who have CCF [7]. Kiuchi K et al. reported CCF leading acute myocardial infarction with coronary steal phenomenon [8]. There is general agreement that symptomatic patients should be treated. All symptomatic patients with coronary artery fistula should undergo closure of the fistula by either surgical or transcatheter approaches. Catheter closure techniques have been performed to treat coronary fistulas with devices, including detachable balloons, stainless steel coils, controlled-release coils, controlled-release patent ductus arteriosus (PDA) coils, and Amplatzer PDA plug [9]. The advantages of the transcatheter approach include less morbidity, lower cost, shorter recovery time, and avoidance of thoracotomy and cardiopulmonary by-pass.

 Hemodynamically significant fistula with a left to right shunt may lead to congestive heart failure, pulmonary artery hypertension, and myocardial ischemia due to a steal phenomenon. The hemodynamic consequence of the coronary cameral fistula depends on the size of the fistula and the communicating chamber. Most coronary artery fistulae are small and usually do not cause any ischemic symptoms and excellent long-term prognosis [10]. López-Candales and Vivek Kumar demonstrated that in patient with coronary artery to left ventricle fistula can be asymptomatic [11]. We present a case of fistula originating from the left anterior descending artery and draining into the left ventricle with two-vessel disease. 

 As a result, in view of the stable angina, absence of heart murmur, and no objective evidence of coronary artery steal, the patient can be managed conservatively.
